# Grain Boundary Wetting by a Second Solid Phase in the High Entropy Alloys: A Review

**DOI:** 10.3390/ma14247506

**Published:** 2021-12-07

**Authors:** Boris B. Straumal, Anna Korneva, Gabriel A. Lopez, Alexei Kuzmin, Eugen Rabkin, Gregory Gerstein, Alexander B. Straumal, Alena S. Gornakova

**Affiliations:** 1 Osipyan Institute of Solid State Physics of the Russian Academy of Sciences, Ac. Osipyan Str. 2, 142432 Chernogolovka, Russia; a.str@issp.ac.ru (A.B.S.); alenahas@issp.ac.ru (A.S.G.); 2 Chernogolovka Scientific Center of the Russian Academy of Sciences, Lesnaja Str. 9, 142432 Chernogolovka, Russia; 3 Institute of Metallurgy and Materials Science, Polish Academy of Sciences, Reymonta St. 25, 30-059 Cracow, Poland; a.korniewa@imim.pl; 4 Physics Department, University of the Basque Country UPV/EHU, Barrio Sarriena s/n, 48940 Leioa, Spain; gabrielalejandro.lopez@ehu.es; 5 Institute of Solid State Physics, University of Latvia, Kengaraga Str. 8, LV-1063 Riga, Latvia; a.kuzmin@cfi.lu.lv; 6 Department of Materials Science and Engineering, Technion–Israel Institute of Technology, Haifa 3200003, Israel; erabkin@technion.ac.il; 7 Institute of Materials Science, Leibnitz University of Hannover An der Universität 2, 30823 Garbsen, Germany; gerstein@iw.uni-hannover.de

**Keywords:** high entropy alloys, grain boundary wetting, precipitation, phase transitions, phase diagrams

## Abstract

In this review, the phenomenon of grain boundary (GB) wetting by the second solid phase is analyzed for the high entropy alloys (HEAs). Similar to the GB wetting by the liquid phase, the GB wetting by the second solid phase can be incomplete (partial) or complete. In the former case, the second solid phase forms in the GB of a matrix, the chain of (usually lenticular) precipitates with a certain non-zero contact angle. In the latter case, it forms in the GB continuous layers between matrix grains which completely separate the matrix crystallites. The GB wetting by the second solid phase can be observed in HEAs produced by all solidification-based technologies. The particle chains or continuous layers of a second solid phase form in GBs also without the mediation of a liquid phase, for example by solid-phase sintering or coatings deposition. To describe the GB wetting by the second solid phase, the new GB tie-lines should be considered in the two- or multiphase areas in the multicomponent phase diagrams for HEAs. The GB wetting by the second solid phase can be used to improve the properties of HEAs by applying the so-called grain boundary engineering methods.

## 1. Introduction

The idea of high-entropy alloys (HEAs) was first proposed in 2004 in seminal works of Cantor et al. and Yeh et al. [1,2]. Usually, HEAs contain at least five different components. One cannot choose the principal one among these components, and the first HEAs were equimolar [1,2,3,4]. Therefore, they are also frequently called multiprincipal component alloys or alloys without principal component. Since 2004, the development of HEAs has been immense. Many thousands of papers are devoted to HEAs. It is impossible to review all aspects of their development. Therefore, this review will be focused on only one important aspect, namely on the phenomena associated with the wetting of grain boundaries by the second solid phase. At the beginning of HEAs studies the important feature of these new materials was that they usually contained only one phase, namely a solid solution where all numerous components were mixed with each other uniformly [1,2,3,4]. Usually, such HEAs have body-centered cubic (bcc) [5,6,7] or face-centered cubic (fcc) [8,9,10,11] lattices. Such homogeneous solid solution is stable in a certain range of compositions and temperatures. Over time, however, researchers’ interest shifted from single-phase HEAs to alloys with numerous elements of heterogeneity. They may include the inhomogeneous distribution of elements in the bulk, the presence of other phases, various grain-boundary layers, etc. This kind of study is called metastability engineering [12,13]. Frequently, the appearance of these heterogeneous structures in HEAs instead of a uniform solid solution can be treated using the ideas of grain boundary (GB) phase transformations. These phase transitions may include the GB wetting with a liquid phase or a second solid phase, as well as the formation of various thin GB layers of the second phase at the boundaries [14,15,16]. Such GB phenomena non-trivially depend on the composition, temperature and pressure in a multicomponent system. In particular, this review paper is devoted to the GB wetting in HEAs with a second solid phase.

## 2. Grain Boundary Wetting by the Second Solid Phase

**Figure 1:**
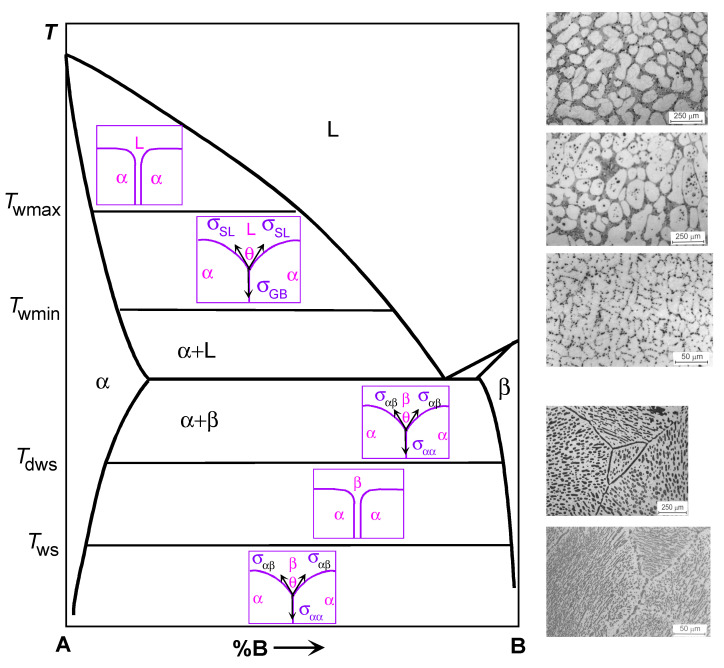
Schematic binary phase diagram for components A and B illustrating the GB wetting phenomena. Bold solid lines show the bulk phase transformation. Thin solid lines show the tie-lines for the GB wetting by the melt at *T*_wmin)_ and *T*_wmax_ as well as by the second solid phase at *T*_ws_ and *T*_dws_. Schemes between solidus and liquidus lines show the cases of complete (top) and partial (bottom) GB wetting by the liquid phase (**L**). Schemes between solvus lines for α- and β-phases show the cases of complete (middle) and partial (top and bottom) wetting of the α/α GB by the second solid phase β. Micrographs on the right-hand side of the diagram (above the dotted line) show the microstructure for the Al–Mg samples annealed above *T*_wmax_ (top micrograph, all GBs are completely wetted), between *T*_wmin_ and *T*_wmax_ (middle micrograph, some GBs are completely wetted and other GBs are partially wetted) and below *T*_wmin_ (bottom micrograph, no completely wetted GBs). Micrographs on the right-hand side of the diagram (above the dotted line) show the microstructure for the Al–Zn samples annealed above *T*_ws_ (top micrograph, all Zn/Zn GBs are completely wetted by the solid (Al) phase) and annealed below *T*_ws_ (bottom micrograph, all Zn/Zn GBs are partially wetted by the solid (Al) phase). Micrographs for Al–Zn alloys are reprinted with permission from Ref. [61]. Copyright 2004 Elsevier.

In the majority of cases, HEAs are manufactured by crystallization from the melt after arc or induction melting [5,6,7,8,9,10,11,17,18,19,20,21,22,23,24,25,26,27,28,29,30,31,32,33,34], electric current assisted sintering [35,36], plasma spark sintering [37], additive manufacturing by the laser powder bed fusion [38,39] or laser metal deposition [40], laser or plasma cladding deposition of coatings [41,42,43,44,45,46,47,48,49,50,51,52], self-propagating high-temperature synthesis (SHS) [53], or even by brazing of dissimilar materials within the brazing joints [54,55]). In some cases, the liquid phase is not present during the synthesis of HEAs such as in the case of sputter deposition of coatings [56] or solid-phase sintering [57]. Figure 1 shows a schematic phase diagram for the simplest case when there are only two components in the studied system. We will apply this schematic phase diagram in further discussion of the GB phenomena. During the cooling of the alloy, it enters first (after crossing the liquidus line) the two-phase L+S region (where L is the melt and S is the solid solution). When the temperature decreases below the solidus line, the melt completely solidifies. The solidification process is much more complicated in the case of HEA. Thus, the six-component alloys need the phase diagram in six dimensions for their description. In this case, there can be not one two-phase region S + L between the single-phase melt L and the single-phase solid solution S, but the regions where several solid and liquid phases can coexist.

Consider now a polycrystalline sample within the two-phase region S + L of the phase diagram. Such a sample should have GBs in the solid phase as well as interphase boundaries (IBs) between the melt and the solid phase. In these alloys the GBs contacting liquid phase form triple junctions with IBs (see Figure 1, inserts in the L field of the phase diagram). Now, if the energy of the two solid/liquid IBs 2σ_SL_ is higher than the GB energy σ_GB_ (lower scheme in area L in Figure 1), then the not zero equilibrium contact angle θ > 0 forms in this TJ is. Such a case with θ > 0 is called partial (or incomplete) GB wetting by the melt. If the energy of two solid/liquid IBs is lower than the GB energy 2σ_SL_ < σ_GB_, then the contact angle should be equal to zero θ = 0. In such a case, the GB would be replaced by a layer of the liquid phase L, and the neighboring grains should be completely separated from each other by a rather thick liquid layer. This is the complete GB wetting by the liquid phase (or melt). Usually, the contact angle θ between the GB and the melt decreases with increasing temperature [58]. In case of a transition from incomplete to complete wetting, the θ reaches zero value at a certain temperature *T*_w_, called the temperature of GB wetting transition. The wetting phase transformation, similar to bulk phase transitions, can be either of first or second order [59]. In the case of a first-order phase transition, the first derivative dθ/d*T* of the contact angle θ with respect to temperature *T* has a discontinuity at *T*_w_ [58,59]. It suddenly changes to zero from a certain finite value [58,59]. If the wetting transition is of a second-order (also called continuous), the dθ/d*T* decreases with increasing temperature continuously and becomes equal to zero at *T*_w_ without a jump at the transformation temperature [59]. The GB energy σ_GB_ strongly depends on the misorientation angle φ and inclination angle ψ of grain boundaries [60]. has sharp cusps at certain φ and ψ values [16]. Obviously, the higher σ_GB_, the smaller the θ at the triple junction of a grain boundary with the melt. The interval of σ_GB_ in polycrystals is very broad. Therefore, at a certain temperature, a wide range of θ values exists in a two-phase polycrystal. With increasing temperature, the contact angles θ for different GBs decrease with different rates. Due to this fact, the *T*_w_ values will be different for GBs with different values of σ_GB_.

On the right hand side of the top of Figure 1, the microstructures of such two-phase binary Al–Mg polycrystals are shown as an example. As a result, two GB wetting border tie-lines appear in the phase diagram. The first line at *T*_wmin_ is for the transition from partial to complete wetting for GBs with the highest σ_GB_ value. Below *T*_wmin_, no completely wetted GBs can be found in a polycrystal. Such polycrystal contains only partially wetted GBs with θ > 0. Above *T*_wmin_, the completely wetted GBs appear in the polycrystal. With further temperature increase, the portion of completely wetted GBs also increases. It reaches unity at *T*_wmax_ indicated in Figure 1 in the (S) + (L) area as a second horizontal tie-line. Above this line at *T*_wmax_, all GBs are completely wetted (see diagram). In this area, each grain “float” in the melt and cannot touch other solid grains. This is because the formation of “dry” GBs is thermodynamically unfavorable since 2σ_SL_ < σ_GB_. Therefore, above the *T*_wmax_ tie-line, all solid grains are separated from each other by rather thick layers (at least a few μm) of the liquid phase. As a result, the new tie-lines for GB wetting phase transition appear in the (S) + (L) two-phase region of a phase diagram. These tie-lines are not represented in the conventional phase diagrams since they ignore the presence of GBs in a system. Accordingly, the microstructure of the polycrystal after solidification will be different for different solidification routes (see dotted lines 1 to 5). It will depend on the path along which the sample crosses the two-phase region during solidification.

Consider now another two-phase region shown at the bottom of the schematic phase diagram in Figure 1. In this two-phase region, two solid phases α and β are in equilibrium. They are two solid solutions, namely that of component (B) in component (A) (α-phase) and component (A) in component (B) (β-phase). There are three types of interfaces in a two-phase polycrystal. First, these are the GBs in the solid solution α, second, these are the GBs in the solid solution β, and third, these are the IBs between the α and β phases. These three types of interfaces can form four types of triple junctions (TJ). First, these are TJs of α/α GBs and TJs of β/β GBs. Second, these are triple junctions of α/α grain boundaries and IBs α/β, as well as TJs of β/β GBs and α/β IBs.

If we analyze the possible relationships of the energies of these grain boundaries σ_αα_, σ_ββ_ and the interface σ_αβ_, then we can also find a situation of complete and incomplete wetting. Thermodynamically, this consideration is very similar to the wetting of GBs by the liquid phase (see above). However, in the case of the coexistence of two solid phases, the wetting phase is also solid. So, let us consider the contact of the GB α/α and the IB α/β. If the grain boundary energy σ_αα_ is less than the energy of two interphase boundaries 2 σ_αβ_, σ_αα_ < 2 σ_αβ_, then a corresponding TJ is characterized by a nonzero contact angle θ > 0 (see the diagram in the lower part of Figure 1). In this case, we have incomplete wetting of the α/α GB by the second phase β. If the energy of two interphase boundaries 2 σ_αβ_ is less than the energy of the grain boundary σ_αα_, σ_αα_ > σ_αβ_, then the α/α GB should be replaced by an interlayer of phase β, and the contact angle will be equal to zero θ = 0. In this case, we are dealing with the complete wetting of the α/α GB by the interlayer of a β-phase.

Until now, we have not seen a qualitative difference between the situation of GB wetting by the second solid phase and by the liquid phase. However, in the liquid phase there are no grain boundaries, while they exist in the second phase β in the considered phase diagram. Therefore, a symmetric situation arises if we consider the grain boundary in the β phase, which is in equilibrium contact with the α phase. If the GB energy σ_ββ_ is less than the energy of two IBs 2σ_αβ_, σ_ββ_ < 2σ_αβ_, then their TJ is characterized by a nonzero contact angle θ > 0 (see the diagram in the lower part of Figure 1). In this case, we have incomplete wetting of the β/β GB by the second solid phase α. If the energy of two IBs 2σ_αβ_ is less than the energy of the grain boundary σ_ββ_, σ_ββ_ > 2 σ_αβ_, then the β/β GB should be replaced by an interlayer of phase α, and the contact angle will be equal to zero θ = 0. In this case, we are dealing with the complete wetting of the β/β GB by an interlayer of the α phase.

Moreover, if the conditions of complete wetting are simultaneously fulfilled for β/β and for α/α GBs, α/α, σ_αα_ > 2σ_αβ_, and σ_ββ_ > 2σ_αβ_, then such GBs cannot exist at all in thermodynamic equilibrium in a two-phase α + β polycrystal. In this case, the two-dimensional section of this polycrystal will topologically resemble a kind of chessboard. In such a polycrystal, there will be no α/α and β/β GBs at all, and only α/β IBs would exist.

There is one more important difference between the GB wetting by the second solid phase and by the melt. In the case of wetting by the liquid phase, the energy of GBs σ_GB_ decreases with increasing temperature, usually more slowly than the energy of the interface between the solid and liquid phases σ_SL_. This is due to the higher entropy of the liquid phase compared to the solid phase. As a result, the contact angle θ always decreases with increasing temperature until it reaches zero value θ = 0 at the transition temperature, after which it remains equal to zero until the sample is completely melted. This behavior of the contact angle has been observed in many binary systems [58,59].

In the case of GB wetting by the second solid phase, there is no such obvious difference in the temperature dependence of the energy of GBs σ_αα_, σ_ββ_ and interphase boundaries σ_αβ_. In this case, the contact angle θ can either increase or decrease with increasing temperature. For example, in the aluminum-magnesium and aluminum-zinc alloys, the behavior of the contact angle was observed, similar to liquid-phase wetting [61]. In this system, the contact angle between (Al)/(Al) GBs and the second solid phase Al_3_Mg decreased with increasing temperature until it reached zero at *T*_ws_, after which complete wetting occurred. In the aluminum-zinc system, on the contrary, the contact angle between (Al)/(Al) GBs and solid zinc decreased with decreasing temperature until it reached zero at the transition temperature *T*_ws_ from incomplete GB wetting to complete one.

Moreover, the temperature dependences σ_αα_ (*T*) and 2σ_αβ_ (*T*) can intersect twice when the temperature changes. In this case, a very interesting phenomenon can be observed, for example, a decrease in the contact angle with an increase in temperature to a certain temperature of the transition to complete wetting *T*_ws_. Then, in a certain temperature range, all GBs in one solid phase are wetted with interlayers in the second solid phase. With a further increase in temperature, a second transition occurs when wetting disappears at the dewetting temperature *T*_dws_. Above this temperature, on the contrary, the GBs in the α phase will be incompletely wetted by the second solid phase β. This phenomenon was observed, for example, in the copper-indium system.

In the schematic Figure 1, in the two-phase region α + β, these tie-lines are shown for wetting at *T*_ws_ and for the dewetting at *T*_dws_. It should be noted here that such a scheme is very simplified. We saw above, when discussing the liquid-phase wetting, that all grain boundaries in a polycrystal have different energies σ_GB_, and for each value of σ_GB_ energy there is its own value of the wetting phase transition temperature *T*_w_. This means that, in reality, the tie-lines at *T*_ws_ and *T*_dws_ should split into a spectrum of lines, and one should draw not one line for each temperature, but at least two lines for the maximum *T*_wsmax_, *T*_dwsmax_ and minimum *T*_wsmin_, *T*_dwsmin_ values of *T*_w_.

Additional complexity in the GB wetting by the solid phase is associated with the fact that, as a rule, the solid-solid IBs are much more anisotropic than their solid-liquid counterparts. Thus, in determining the value of contact angle θ not only the GB and IB energies, but also their angular derivatives (Herring’s torque terms) should be taken into account [62]. The amplitude of the latter decreases with increasing temperature.

Until now, we have spoken about the thermodynamic conditions of complete and incomplete GB wetting in one solid phase by the interlayers of another solid phase. However, there is also an important kinetic difference. In the case of wetting with the liquid phase, the equilibrium is reached very quickly due to the high rate of mass transfer in the melt. Usually, the half-an-hour long annealing is fully enough for thermodynamic equilibrium to occur and equilibrium values of the contact angle è of GBs with the liquid phase are established. In the case of wetting with the second solid phase, the mass transfer is much slower. Therefore, in experiments on GB wetting with the second solid phase, much longer annealing durations should be used, which can last for many months to reach the thermodynamic equilibrium.

Now we discuss two groups of the results on GB wetting by the second solid phase. (1) The examples where this phenomenon is observed in primarily inhomogeneous HEAs. These HEAs were produced by crystallization from the melt, and the GBs were wetted by the liquid phase during solidification, and an inhomogeneous structure formed. Afterwards these alloys were annealed in the solid state, and primary inhomogeneity was retained. The reason for such inhomogeneity may be possible equilibrium GB wetting by the second solid phase. (2) The cases where the GB wetting by second solid phase was observed in primarily homogeneous HEAs. It means the layers of the wetting GB phase appear during precipitation annealing of primarily homogeneous HEAs.

## 3. GB Wetting by Second Solid Phase in Primarily Inhomogeneous HEAs (after Solidification and Following Short Annealing)

In Ref. [9] the Al_0.5_CoCuNiTi HEA was prepared by arc-melting. In the as-cast state, they are composed of the fcc matrix grains separated by the 1–4 µm thick layers of the bcc phase. The bcc GB phase is enriched in copper. It is also well visible from the micrographs that not all GBs were completely wetted by the liquid phase. Some of the GBs remained incompletely wetted with a non-zero contact angle. The additional annealing at 800 °C for 20 h did not qualitatively change the microstructure. There are two possible explanations for this fact. First, the bcc phase wets GBs in the fcc matrix also as a second solid phase. Second, the annealing duration was not long enough to reach the equilibrium. The annealing in the works devoted to the GB wetting of the second solid phase was much longer, up to 5000 h.

**Figure 2:**
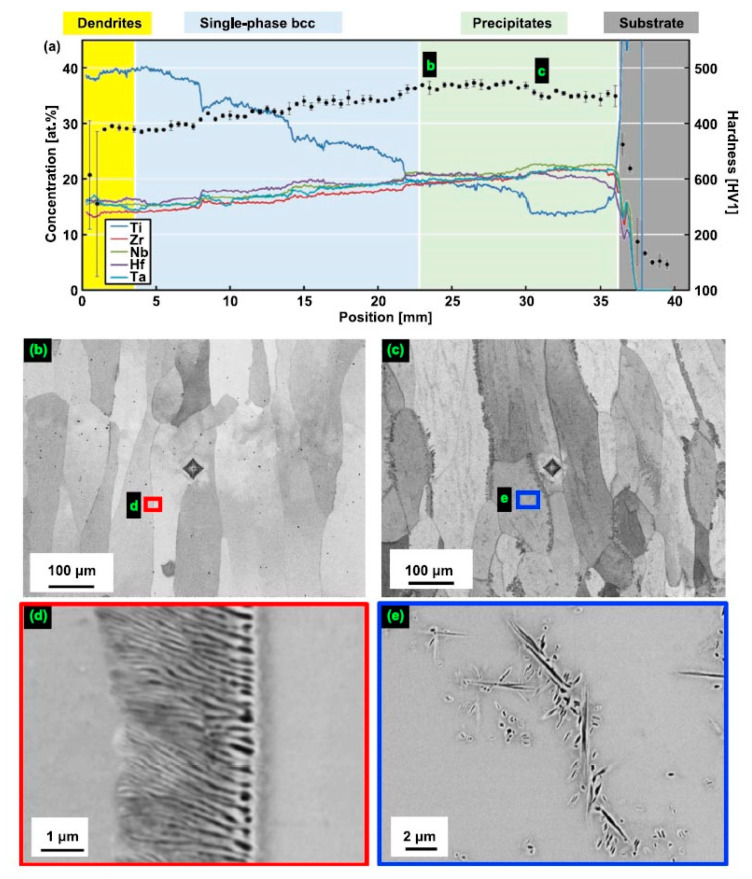
Microstructure of compositionally graded sample built using a blend of five elemental powders [63]. (**a**) The composition was changed from Ti_13.3_Zr_21.7_Nb_21.7_Hf_21.7_Ta_21.7_ (right) to Ti_40_Zr_15_Nb_15_Hf_15_Ta_15_ (left). The position *x* = 0 indicates the top of the columnar specimen while positions to the right of *x* = 36 mm are located within the Ti substrate. The concentrations of the alloying elements (left *y*-axis) are shown as continuous lines, and hardness values (right *y*-axis) are given with black dots and error bars. The labels (**b**,**c**) indicate the positions where the microstructure was investigated by SEM and found to exhibit two phases. In figures (**b**,**c**), small areas marked with colored frames are magnified in (**d**,**e**), respectively. Lamellar precipitates were found at grain boundaries (**b**–**e**). Additionally, plate-shaped precipitates formed within grains at location (**c**,**e**). Reprinted with permission from Ref. [63]. Copyright 2021 Elsevier.

The TiZrNbHfTa gradient HEAs were produced in Ref. [63] by the laser metal deposition (LMD) of elemental powder blends on a 3 mm-thick Ti substrate using a fiber laser (Manlight Gevel, Lannion, France) with a spot diameter of ~5 mm and a gaussian beam profile. The manufactured samples were annealed in the solid state which homogenized the dendritic microstructure of the deposited layers. In Figure 2 the microstructure of the compositionally graded sample built using five powder blends from the powder is shown [63]. The composition was changed from Ti_13.3_Zr_21.7_Nb_21.7_Hf_21.7_Ta_21.7_ (close to the substrate) to Ti_40_Zr_15_Nb_15_Hf_15_Ta_15_ (top of the deposited layer) (Figure 2a). Lamellar precipitates were found at grain boundaries Figure 2b–e. Additionally, plate-shaped precipitates formed within grains at location Figure 2c,e. The matrix possesses the bcc structure, the crystallographic structure of precipitates was not determined [63]. The two types of precipitates in figure Figure 2d,e were simultaneously enriched in Zr, Hf, O (the EDX peaks of O and N cannot be differentiated) and depleted in Ti and Ta. The morphologies of these latter precipitates are similar to those reported by Senkov and Semiatin for an equiatomic TiZrNbHfTa RHEA that was annealed at 1200 °C for 2 h and slowly cooled at a rate of 15 °C/min to room temperature. One can interpret also the structures shown in Ref. [5] as incomplete wetting of GBs in the bcc matrix by the second solid phase since the GB precipitates do not from the continuous layers.

The CrHfNbTaTi high-entropy alloy, derived by replacement of Zr by Cr in HfNbTaTiZr has been prepared by arc melting in a high-purity Ar atmosphere [10]. Following annealing treatment was carried out under vacuum condition at 1273 K for 20 h. The matrix consisted of the bcc phase. In the as-cast state, almost all grains of the matrix bcc phase are separated from each other by the 25 μm thick layers of Cr,Hf-rich fcc Laves phase of C15 type. This structure shows that during solidification the GBs in the matrix bcc phase were completely or partially wetted by the melt rich in Cr and Hf. After annealing treatment in the solid-state at 1273 K for 20 h the concentration differences become weaker, however, the layers of the fcc Laves phase still separate almost all grains of the bcc phase. It can be the indication that GBs in the bcc phase can be wetted by the fcc Laves phase of C15 type also in the solid-state. This fact can be proved by the longer anneals at different temperatures.

Similar behavior has been observed in the TiNbTaZrMo alloy prepared by the vacuum arc melting and subsequent annealing at 1200 °C for 168 h [64]. In the as-cast state as well as after annealing the alloy contained two bcc phases, bcc-1 and bcc-2. The Zr- and Nb-rich layers of the bcc-2 phase completely separated about half of the bcc-1 matrix grains (complete GB wetting). Another half of bcc-1/bcc-1 GBs were partially wetted. As in Ref. [64], such structure indicates the GB wetting by the melt and may indicate also the presence of GB wetting by the second phase bcc-2 in the solid-state.

**Figure 3:**
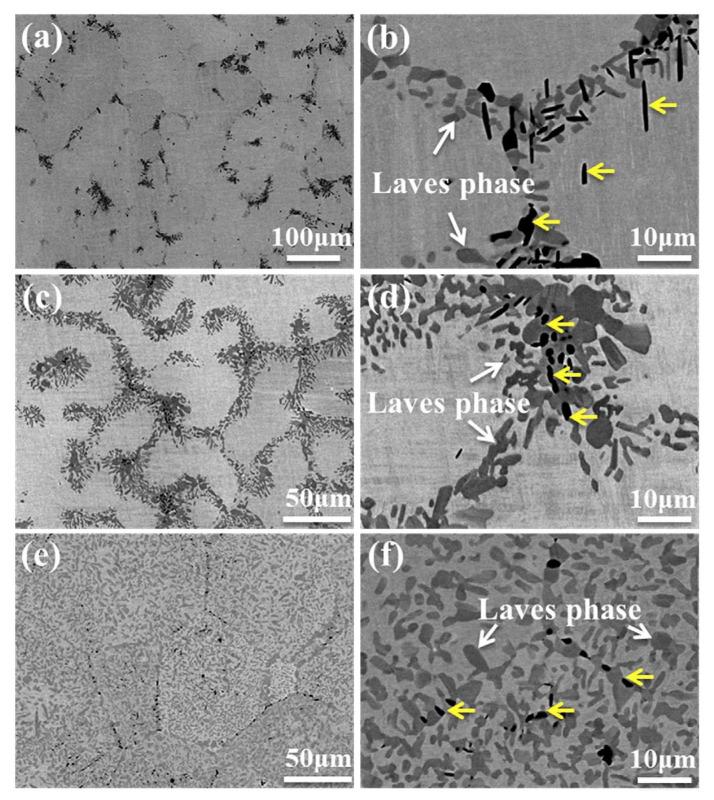
SEM backscatter electron images of the annealed (**a**,**b**) Cr1.0, (**c**,**d**) Cr1.5 and (**e**,**f**) Cr2.0 alloys. Reprinted with permission from Ref. [26]. Copyright 2020 Elsevier.

**Figure 4:**
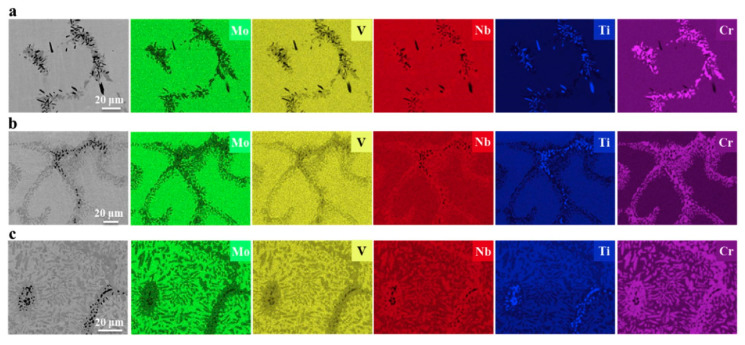
SEM/EDS mappings of Mo, V, Nb, Ti and Cr in the annealed (**a**) Cr1.0, (**b**) Cr1.5 and (**c**) Cr2.0 alloys. Reprinted with permission from Ref. [26]. Copyright 2020 Elsevier.

The Mo_0.5_VNbTiCr_x_ (with *x* = 0−2.0) HEAs were produced by vacuum arc melting, followed by hot isostatic pressing at 1200 °C and 150 MPa for 2 h and annealing at 1200 °C for 72 h [26]. The hot isostatic pressing of the samples implies that the alloy “forgets” the solidification microstructure. Thus, the following rather long annealing may enable reaching the thermodynamic equilibrium is reached for 1200 °C. The resulted microstructure consists of the matrix grains with bcc lattice and GB particle chains or layers of two phases, dark grey (yellow arrows) and light grey (white arrows), respectively (Figure 3). The light grey phase (Laves phase) is rich in Cr and Nb, and the dark phase is heavily enriched with Ti (Figure 4). The dark phase forms only chains of precipitates (partial GB wetting) and Laves phase can form chains of precipitates (partial GB wetting, at low Cr content) as well as continuous GB layers (complete GB wetting, at high Cr content) (Figure 3). It is not surprising because the addition of chromium decreases the lattice period in the matrix bcc phase [26]. Therefore, the varying Cr content influences also the thermodynamic conditions for the GB wetting (i.e., the GB σ_αα_ and IB σ_αβ_ energies).

The Co_35_Cr_32_Ni_27_-Al_3_Ti_3_ (at.%) HEA was cast by a vacuum induction melting method under a pure Ar atmosphere [17]. After homogenizing annealing at 1200 °C for 2 h, the ingot was forged repeatedly at about 1150 °C with an area reduction of 80%. After that, the alloy was subsequently annealed at 700 °C for 8 h followed by water quenching. It contains the fcc matrix and L1_2_ and hcp precipitates in GBs. The from the chains of elongated GB particles (incomplete GB wetting).

Eight V–Cr–Mn–Ti alloys were prepared by arc melting process in an argon atmosphere [40]. These samples were then homogenized at 1200 °C for 100 h and quenched in water. The matrix appeared as a single bcc phase in all alloys. The precipitates were Ti-rich and have an fcc structure. They have rounded shapes and form chains in GBs (incomplete wetting). The amount and shapes (i.e., contact angles θ) of precipitates are different for different compositions.

The AlCoCrFeNiTi_0.5_ HEA ingots were prepared by the arc melting method in the argon atmosphere [65]. The as-cast state contained the simple solid solution based on bcc as the main phase. The specimens were then annealed at 800 °C for 5 h. They contained additionally the fcc phase in GBs separating the matrix grains. The fraction of GBs completely and partially wetted by the fcc phase can be estimated as 50:50.

Thus, there are many examples where the GB wetting by the second solid phase was observed in primarily inhomogeneous HEAs. These HEAs were produced by crystallization from the melt. During solidification, the GBs were wetted by the liquid phase, and an inhomogeneous structure formed. Afterward, these samples were annealed in the solid-state, but the homogenization was not completed, and primary inhomogeneity was retained. It means that the equilibrium GB wetting by the second solid phase can take place.

## 4. GB Wetting by Second Solid Phase in Primarily Homogeneous HEAs (after Precipitation Annealings)

More convincing examples of the GB wetting by the second solid phase can be found when the starting state of the samples is homogeneous. When one anneals such samples in the solid-state long enough, and as a result of such anneals the GB layers of the second solid phase (precipitates) appear and separate the abutting grains, one can confidently speak about the equilibrium phenomenon of GB wetting by the second solid phase. 

**Figure 5:**
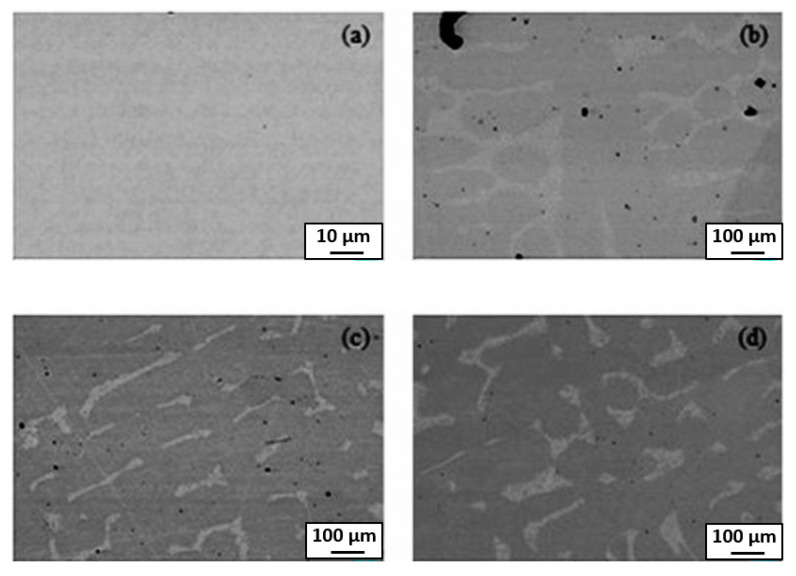
BSE-SEM pictures of (**a**) homogenized and quenched sample (CoCrFeMnNi)_100-5_Ti_5_ as well as the samples following aged at 750 °C (**b**) 2 h, (**c**) 10 h and (**d**) 20 h. The images are not from the same spot and were obtained from different samples. Reprinted with permission from Ref. [37]. Copyright 2021 Elsevier.

**Figure 6:**
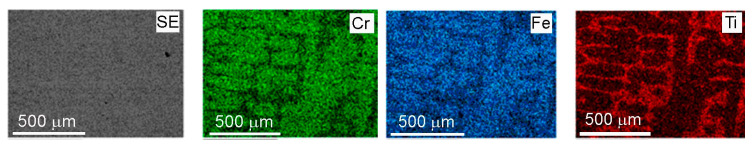
EDX-mapping of homogenized and quenched sample (CoCrFeMnNi)_100-5_Ti_5_. Reprinted with permission from Ref. [37]. Copyright 2021 Elsevier.

The alloys, which are based on Cantor HEA CoCrFeMnNi and additionally alloyed with 2–5 wt.% Ti, have been plasma spark sintered in argon from elementary powders at 1000 °C (i.e without melting), then melted at 1400 °C and finally solution annealed under air (1250 °C, 1 h) [37]. In this state, the alloys were completely homogenous and contained one fcc phase (Figure 5a). The following precipitation heat treatment was conducted at 650, 750 and 850 °C for 2 to 20 h. As a result, the 10–30 µm thick layers of the Ti-rich phase formed along the GBs of the fcc matrix phase (Figure 5b–d). In Figure 5b–d it is well visible how the GB layers appear and grow with increasing annealing duration from 2 to 20 h at 750 °C. Not all GBs have such a layer of Ti-rich phase. It means that only about 50% of GBs in the fcc matrix are completely wetted by a second Ti-rich phase (Figure 6). Other GBs are partially (or incompletely) wetted (see scheme in Figure 1).

**Figure 7:**
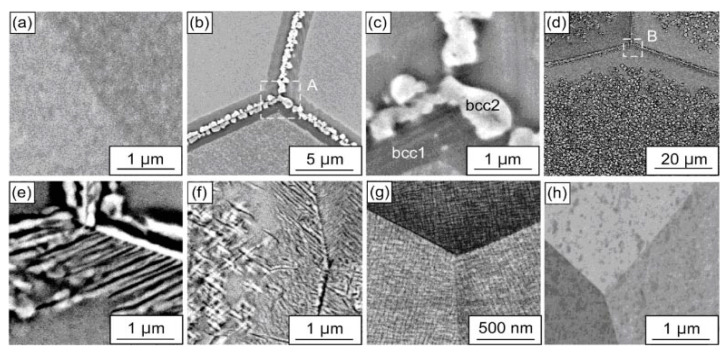
BSE images of the HfNbTaTiZr polycrystals annealed at 673–1173 K for 24 h. (**a**) 1173 K, (**b**,**c**) 1073 K, (**d**,**e**) 973 K, (**f**) 873 K, (**g**) 773 K and (**h**) 673 K. (**c**,**e**) are enlarged views of regions A and B in (**b**,**d**), respectively. Reprinted with permission from Ref. [5]. Copyright 2021 Elsevier.

In the case of the equiatomic HfNbTaTiZr high entropy alloy prepared by the plasma arc melting the two-phase S + L area of the phase diagram does not contain any tie-lines of the GB wetting transition and the one-phase solid solution polycrystal with typical dendritic structure forms after solidification [5]. After homogenization at 1473 K, this dendritic structure disappears and the bcc solid solution becomes homogenous (Figure 7a). The following anneals show that at 1173 K the HfNbTaTiZr HEA is still a single phase. However, at 1073 K the first precipitates of the second phase bcc-2 appear. They form almost continuous 500–900 nm thick layers along the GBs in the bcc-1 matrix (Figure 7b,c). It means that the second solid phase bcc-2 completely wets the GBs in the bcc-1 matrix phase. At 973, 873 and 773 K the third phase with hcp structure appears in the XRD patterns. The bcc-2 and hcp phases also completely wet the GBs in the bcc-1 matrix at 973 and 873 K (Figure 7d–f). At 773 K the bcc-2 and hcp phases are present in XRD patterns, but they do not form the continuous GB layers and are present only in the bulk (Figure 7g). It means that between 873 and 773 K the dewetting of the GBs of the bcc-1 phase takes place. At 673 K the bcc-2 and hcp phases completely disappear from XRD patterns and are absent both in the bulk and at the GBs Figure 7h). Thus, the GBs in the bcc-1 phase are completely wetted by the second solid phase(s) only in the certain temperature interval between 773 and1073 K. This phenomenon is similar to wetting and dewetting of GBs in (Cu) solid solution by the solid δ-phase in the Cu–In alloys. 

The four-component Ti–29Nb–13Ta–4.6Zr alloy known also as TNTZ (such alloys are frequently called medium-entropy alloys) was melted in a vacuum [21]. The ingot was solution treated under a temperature of 1073 K for 2 h, in order to eliminate the casting defects such as component segregation. After this annealing, the alloy contained mainly the bcc β-phase and a few percent of hcp α-phase. The α-phase formed the perfect few μm thin intergranular layers between the matrix β-grains. It means that in the four-component β-Ti TNTZ alloy the solid α-phase can completely wet the β/β GBs, similar to our observations in the binary and ternary titanium and zirconium alloys with α + β structure.

**Figure 8:**
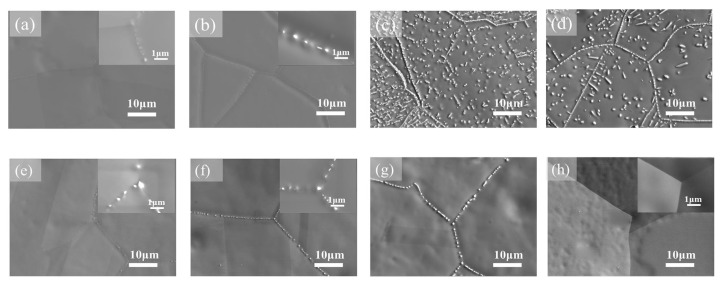
SEM images of precipitates in the (Co_35_Cr_25_Fe_40-*x*_ Ni*_x_*)_94_Ti_3_Al_3_ with *x* = 5 (**a**–**d**) and (Co_35_Cr_25_Fe_40-*x*_ Ni*_x_*)_94_Ti_3_Al_3_ with *x* = 14 (e–h) alloys aged for 1 h at temperatures of (**a**,**e**) 700 °C, (**b**,**f**) 800 °C, (**c**,**g**) 900 °C, and (**d**,**h**) 1000 °C. Reprinted with permission from Ref. [66]. Copyright 2019 Elsevier.

**Figure 9:**
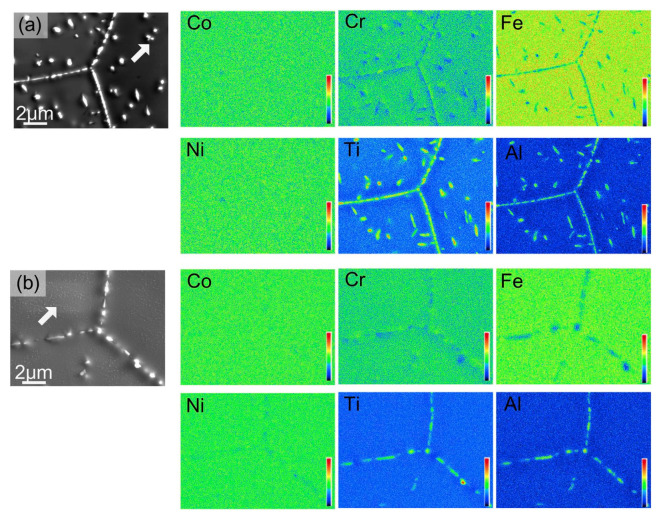
Elemental mapping of (Co_35_Cr_25_Fe_40-*x*_ Ni*_x_*)_94_Ti_3_Al_3_ with *x* = 5 (**a**) and (Co_35_Cr_25_Fe_40-*x*_ Ni*_x_*)_94_Ti_3_Al_3_ with *x* = 14 (**b**) alloy after aging at 800 °C for 16 h. Reprinted with permission from Ref. [66]. Copyright 2019 Elsevier.

The HEAs Co_35_Cr_25_Fe_40-*x*_Ni*_x_* (*x* = 5 and 14 at. %, named as Ni5 and Ni14) and (Co_35_Cr_25_Fe_40-*x*_ Ni*_x_*)_94_Ti_3_Al_3_ (*x* = 5 and 14 at. %, named as Ni5-TA and Ni14-TA) were prepared by arc-melting mixtures of the constituent elements [66]. The as-cast ingots were homogenized at 1200 °C for 4 h, and then cold rolled. Afterward, the samples were annealed at 1150 °C for 5 min and quenched in water. After this homogenization, the specimens have been aged at 700, 800, 900 and 1000 °C between 1 h and 16 h with the following water quenching. This complicated experimental procedure implies to us that the thermodynamic equilibrium was reached at 700, 800, 900 and 1000 °C. XRD shows that the samples consist of an fcc matrix and a small amount of hcp phase. SEM demonstrates that the hcp phase is distributed mainly in fcc/fcc GBs (Figure 8). In the (Co_35_Cr_25_Fe_40-*x*_ Ni*_x_*)_94_Ti_3_Al_3_ with *x* = 5 alloy the hcp phase forms the particle chains (incomplete wetting) at 700 °C (Figure 8a) and 800 °C (Figure 8b). At 900 and 1000 °C appear the matrix GB completely “wetted” by the hcp phase (Figure 8c,d). The alloys (Co_35_Cr_25_Fe_40-*x*_ Ni*_x_*)_94_Ti_3_Al_3_ with *x* = 14 behave differently. At 700, 800 and 900 °C we observe only the chains of precipitates in GBs and, therefore, the partial GB wetting (Figure 8e–g). At 1000 °C the second phase with hcp lattice is completely absent both in bulk and in GBs (Figure 8h). The elemental mapping shows that the GB wetting phase is enriched by Ti and Al (Figure 9). Thus, here we also can see that the change of HEA composition modifies the conditions for the GB wetting and, therefore, the morphology of GB precipitates.

**Figure 10:**
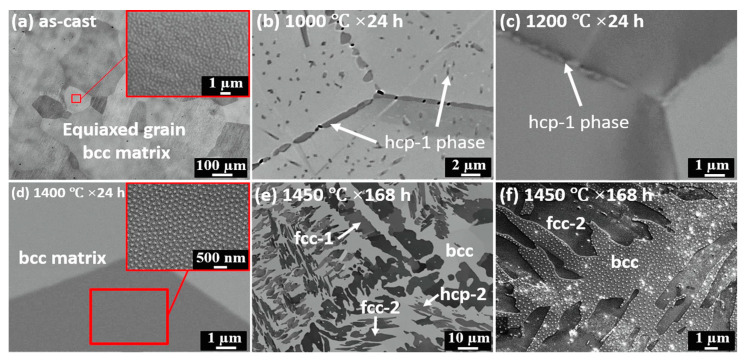
Microstructure evolution of HfNbTaTiZr RHEA at various conditions: (**a**) as-cast; (**b**) 1000 °C, 24 h; (**c**) 1200 °C, 24 h; (**d**) 1400 °C, 24 h; (**e**) 1450 °C, 168 h; (**f**) a magnified image of observed precipitates in condition of 1450 °C, 168 h. Reprinted with permission from Ref. [67]. Copyright 2019 Elsevier.

**Figure 11:**
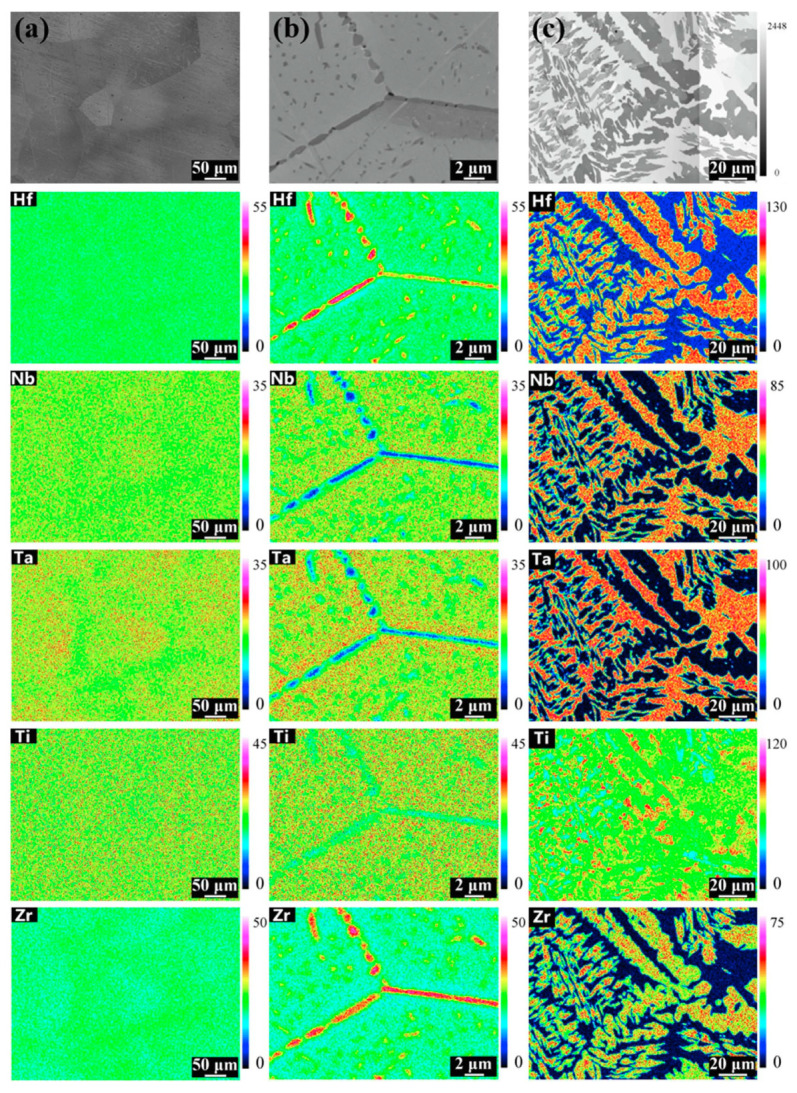
Elemental mapping at different conditions: (**a**) as-cast; (**b**) 1000 °C, 24 h; (**c**) 1450 °C, 168 h. Reprinted with permission from Ref. [67]. Copyright 2019 Elsevier.

The perfect example of incomplete and complete GB wetting by the second solid phase in HEAs can be found in Ref. [67]. The equimolar HfNbTaTiZr HEA was prepared by arc melting under argon. The samples were then annealed at 1000 °C, 24 h; (c) 1200 °C, 24 h; (d) 1400 °C, 24 h; (e) 1450 °C, 168 h with following furnace cooling. The as-cast alloy contains only one bcc phase, without any precipitates (Figure 10a and Figure 11a). After annealing at 1000 °C the hcp-1 phase appears in the alloy (Figure 10b and Figure 11b). It is enriched on Hf and Zr (Figure 11b). The hcp-1 phase is positioned mainly in GBs and few precipitates are present also in the bulk. The micrograph in Figure 10b shows the GB TJ and perfectly all three major GB wetting cases. The GB on the right-hand side is completely wetted by the solid hcp-1 phase which forms the continuous layer of uniform thickness separating the bcc grains. The GB in the upper part of the micrograph contains the chain of the hcp-1 separated by the “dry” GB. Remarkable is the similar shape of all precipitates. They demonstrate with almost the same contact angle with “dry” GB portions. The GB on the left-hand side is the intermediate case. Most probably, the condition of complete GB wetting is only partially fulfilled for this GB. As a result, the GB portion close to the triple junction is completely wetted. Far away from TJ the continuous GB layer breaks and partial GB wetting is observed. A similar dependence of GB wetting on the GB inclination we observed in binary Al–Zn alloy [61]. At 1200 °C the complete and partial GB wetting by hcp-1 phase are still observed (Figure 10c). At 1200 °C the hcp-1 phase disappears both from bulk and GBs. At 1450 °C appear the fcc-1, fcc-2 and hcp-2 phases, and the morphology of precipitates becomes very complicated (Figure 10e,f and Figure 11c)

Elemental powders were used to prepare TiNbTa_0.5_ZrAl_0.5_ HEA through the PM method [68]. The compacts were sintered at 1300 °C for 16 h in a vacuum with following furnace cooling. The samples were then hot forged at 1200 °C with a 50% reduction followed by furnace cooling. Thus, the solidification of the fully liquid samples was excluded. In the as-sintered state, the alloys contained a single phase of bcc crystal structure. After hot forging the (Zr, Al)-rich precipitates with hcp structure appear in the alloy. The analysis of the figures from Ref. [68] shows that about one-third of GBs in the matrix bcc phase are completely wetted by the uniform layers of hcp (Zr, Al)-rich phase. Other GBs contain the chains of (Zr, Al)-rich precipitates, i.e., they are partially wetted.

Co-Ni-Al-W-Ta-Ti senary alloys were prepared by arc melting under argon atmosphere [69]. Solution heat treatment in sealed quartz tubes back-filled with Ar was conducted at 1270 °C for 24 h, and long-term aging treatments were subsequently conducted at 1100 °C and 1150 °C for 1000 h, followed by water quenching. All alloys contain the typical for superalloys γ + γ′ phase mixture. exhibited a γ + γ′ two-phase microstructure with no precipitation of other secondary phases. 

In Ref. [70] the equiatomic CoCrFeMnNi HEA were prepared by the vacuum induction melting. In the as-cast state, they have a uniform structure and contain only one fcc phase. Afterwards the samples were not simply annealed but hot-deformed at 700 °C. The samples were uniaxially compressed to 70% at a strain rate of 0.01/s. During this thermomechanical treatment, the precipitation of Cr-rich σ-phase took place. The σ-phase precipitates were observed only in GBs of the fcc matrix. They did not form the continuous GB layers. In other words, σ-phase only partially wets the fcc/fcc GBs. One can see in micrographs in Ref. [70] that σ-precipitates have different contact angles in different GBs. It is due to the difference in GB energy for GBs with various characters.

In Ref. [71] the medium-entropy alloy with a nominal composition of Al_7.45_(CoCrNi)_92.55_ (at.%) was fabricated using an arc-melting furnace in an argon atmosphere. The as-cast alloy was homogenized at 1100 °C for 24 h. Subsequently, the specimen was cold rolled with a total thickness reduction of 90%. Then the alloy was annealed at 1100 °C for 5 min and followed by water quenching to obtain the recrystallized structure with a single-phase. The following heat treatment process was carried out at 700, 800, and 900 °C for 10 h to promote the second-phase precipitation. After second annealing at 1100 °C and water quenching the alloy contained only one fcc phase. During heat treatments at 700, 800, and 900 °C the precipitates of the B2 phase formed in almost all GBs. The majority of fcc/fcc GBs was incompletely wetted by the B2 phase. However, some matrix GB contained continuous layers of the B2 phase demonstrating the complete GB wetting by a second solid phase.

In this paper, we gave important examples of GB wetting by the second solid phase in HEAs. These examples do not exhaust all cases of such GB wetting in HEAs. They are just typical ones selected from recent publications. Indeed, similar microstructures can be frequently seen in the publications devoted to HEAs. Therefore, we can see that advanced HEAs may contain more than one homogeneous solid solution phase. The morphology of the minor phase(s) in such homogeneous HEAs can depend on the phenomena of GB wetting by the second solid phase. Such GB wetting can control the distribution of the minor phase between the grains of the major phase. Recently, such phenomenon has been studied in binary metallic alloys. The obtained data can be effectively used also for the description of multicomponent HEAs. In particular, in the two- and multiphase areas of a multicomponent phase diagram can appear the additional tie-lines of the GB phase transitions, for example at wetting *T*_ws_ and dewetting *T*_dws_ temperatures (see Figure 1). As a result, the minor second solid phase can form the quite thick (some μm) layers which separate the grains of the major phase from each other. Such GB layers may have both positive and negative effects on the HEA properties. For example, they can improve the mechanical properties of HEAs and their thermal stability by stabilizing the grain structure and preventing the grain growth at elevated temperatures. On the other hand, if the GB phase is brittle, one needs to prevent the complete GB wetting. Otherwise, the continuous GB layers can lead to brittleness of the whole material (for example GB cementite in wrongly treated high-carbon steels). One can apply the knowledge on the GB wetting transformations by the second solid phase in HEAs for tailoring their properties and the further development of these important materials.

We have also to underline here one important point. We discussed in this short review only bulk materials where GBs are wetted by the “true” three-dimensional phases and all samples are rather thick. However, we observed previously in binary alloys, that GBs can also contain the few nm thin layers of a second phase [15]. Such layers consist of a phase that is stabilized in a GB but are unstable in the bulk. They appear during GB phase transitions such as premelting, prewetting of pseudoincomplete wetting [15]. Similar phenomena should definitely be present also in HEAs. Especially promising from this point of view are HEA thin films [72,73,74,75]. The HEA thin films are usually nanogranied and have, therefore, a high specific density of GBs. On the other hand, the ultrafine grained bulk HEAs can be produced, for example, by the so-called severe plastic deformation (SPD) [76,77,78]. There are at least hundreds of publications devoted to nanograined HEA thin films and SPD of HEAs. We plan to describe the respective GB phenomena in future reviews.

## 5. Conclusions

The thick (some μm) grain boundary layers of a second solid phase can appear in HEAs during annealing. The formation of such thick GB layers is due to the phenomenon of complete or incomplete GB wetting by a second solid phase. Thermodynamically, it is similar to the GB wetting by a melt. Kinetically, one needs much longer annealing times to reach equilibrium in the case when the wetting phase is solid. Thus, the equilibrium solid layers between matrix grains are formed when the GB energy is higher than the energy of two interphase boundaries. The presence of GB layers of a second phase can be either positive or detrimental for the properties of HEAs. In any case, one can use the phenomena of GB wetting by a second solid phase to tailor the microstructure and properties of HEAs. For such so-called grain boundary engineering of HEAs one needs knowledge about the position of GB wetting tie-lines in the bulk phase diagrams of HEAs.

## Data Availability

Data is contained within the article.
